# Estimation of an Upper Bound to the Value of the Step Potentials in Two-Layered Soils from Grounding Resistance Measurements

**DOI:** 10.3390/ma13020290

**Published:** 2020-01-08

**Authors:** Jorge Moreno, Pascual Simon, Eduardo Faleiro, Gabriel Asensio, Jose Antonio Fernandez

**Affiliations:** 1 Polytechnic University of Madrid (UPM), Escuela Técnica Superior de Ingeniería y Diseño Industrial (ETSIDI), Ronda de Valencia 3, 28012 Madrid, Spain; jorge.moreno@upm.es (J.M.); gabriel.asensio@upm.es (G.A.); 2 LCOE Laboratory, Calle Diesel 13. P.I. El Lomo, 28906 Madrid, Spain; psimon@ffii.es; 3 Union Fenosa Distribución (UFD). Avenida San Luis 77, 28033 Madrid, Spain; jafernandez@ufd.es

**Keywords:** grounding electrodes in two-layered soils, step and touch potentials, step potentials upper bound

## Abstract

Due to the constant updating of regulatory standards on safety issues in electrical installations, limits are established for the maximum step potential that an installation can hold in a ground fault situation. In this paper, an upper bound to the maximum value of the step potentials arising in the soil surface when a fault takes place in a grounded electrical installation is estimated by means of a simple procedure. The direct measurement of the grounding electrode resistance together with some information about the soil resistivity and the knowledge of characteristic parameters of the electrode are used for the calculation of that upper bound. The procedure is tested at numerical simulation level by using different electrodes in several different scenarios corresponding to two-layered soils with different resistivity ratios. The dependency of the calculated upper bound with the electrode burial depth is also studied. Finally, a real case study is presented, and the results of the field measurements are shown as an example of the validity of the procedure.

## 1. Introduction

All electrical facilities such as transformation centers substations or transmission towers need to have a ground protection system installed that guarantees the evacuation to the ground of fault currents that could otherwise seriously damage people and the facility itself. For this purpose, a metallic electrode is buried into the ground which receives and disperses the fault currents by raising its own electric potential and that of its surroundings. This is the so-called grounding electrode.

When a fault takes place in a facility equipped with a grounding electrode, the surface of the ground experiences a rise in electrical potential that can put people and equipment at risk in its area of influence [1]. Among the magnitudes most frequently used to quantify the effect of a ground fault in an installation are the step potentials and the touch potentials. The touch potential is the potential difference between the grounding electrode and some point on the ground surface. The step potential is the potential difference between two points of the ground separated by a distance of one meter, and it is a measure of the gradient of the absolute potentials generated in the soil. The step potential reaches maximum values at the ground points near the buried electrode, that is, areas in which technical staff may be working.

The authorities responsible for ensuring the safety of electrical installations establish maximum values for the step and touch potentials that must be met in case of fault. The European Union, for example, establishes general regulations that each member country adapts to its own standards [2,3]. These potentials should be measured directly in the field unless there is an indirect procedure that ensures compliance with the maximum values officially established without the need to be directly measured. Having such a procedure not only means a considerable economic saving given the cost of direct measurement on the ground but also a considerable simplification when the step potentials must be estimated in urban areas where direct measurement is often difficult. From decades, many authors have spent great efforts to propose calculation methods for step potentials, from the exclusive use of approximate analytical formulas [4,5] to using complex numerical techniques [6], while they were measured in the field by using various strategies [7,8]. However, none of the methods known to the authors of this paper is as simple and quick to apply as the one proposed here [9].

The motivation of the present work is in part of an economic nature. Regulations regarding safety in electrical installations are constantly being updated. The tolerance with respect to step and touch potentials is frequently reviewed, so it is necessary to check if the facilities comply with the updated regulatory framework. As stated before, direct measurements in the field are usually expensive, so an estimation procedure that can avoid such measurements will always be welcomed by electric companies.

In the present paper, a procedure for estimating an upper limit to the value of the step potentials generated at the ground surface by a grounding electrode driving a fault current to ground is proposed. The comparison of such an upper limit with the maximum regulatory values for the installation will allow discarding the direct measurement if these values are above the obtained limit. Otherwise, a direct measurement will be necessary to decide whether the grounding system complies with the regulation [10].

The procedure is based on the maximization of the quotient of two characteristic parameters of the grounding electrode involving the resistance of the electrode and the normalized step potentials that it generates in the soil surface when a fault takes place. 

To make the procedure known in detail, the paper is organized as follows. After the present introduction, in Section 2 of the paper, the fundamentals of the procedure are detailed, and in Section 3, the procedure is tested in some numerical experiments by using two-layered soils. In Section 4, we present a real case of study to which the procedure is applied and finally, in Section 5, the conclusions of the paper are collected.

## 2. Basics of the Procedure

The procedure is based on establishing a relationship between the step potentials generated by an energized grounding electrode and the potential acquired by the electrode itself in that situation. Both potentials are expressed as a function of the coefficients *K_r_* and *K_p_*, which are defined as

(1) $$K_{r}=\frac{U_{r}}{\rho_{r}\cdot I}\;K_{P}=\frac{U_{P}}{\rho_{P}\cdot I}$$

where *U_r_* and *U_p_* are the potential of the electrode and the maximum step potential generated in the soil surface when a current *I* is leaked to the ground, respectively. The resistivities *ρ_r_* and *ρ_p_* correspond to the equivalent resistivity of the soil at the depth of the electrode and the equivalent resistivity at surface level, which is where the step potentials are measured, respectively. Equivalent resistivities are not true resistivities in general but represent the resistivity that a homogeneous soil would have, so that the potential generated by the electrode in such soil coincides with the value of the potential in the true soil. Regarding the step potential, for each electrode, the procedure to calculate this potential is usually given as a standard rule and generally corresponds to the highest possible value among those evaluated at locations accessible by the company staff who may be near the electrode when a fault event takes place. According to the type of electrode, it is frequent that the procedure to calculate the maximum step potential is prefixed by the manufacturer in the specifications of the electrode itself. The calculations of *K_r_* and *K_p_* are made by calculating the potential acquired by the electrode and the maximum step potential created in the soil surface according to the electrode specifications, when both the resistivity and the electric current are of unit value. This calculation is usually carried out by means of a computer simulation in a homogeneous semi-infinite soil where the electrode is placed in the position and at the depth that it will really have. The simulation method is the one commonly used in this type of problems, that is, boundary elements together with the thin wire approach for the electrodes and the method of moments for the numerical solution of Maxwell’s equations with boundary conditions only at the ground surface [9]. The main Spanish Electric Companies, for example, have an extensive database with the specifications of the grounding electrodes used in their facilities, among which are the values of *K_r_* and *K_p_*. These parameters may have been supplied by the manufacturers or are instead theoretically calculated from the knowledge of the shape and size of the electrodes as has been done in this paper.

From the expressions (1) it is obtained

(2) $$\frac{U_{p}}{U_{r}}=\frac{K_{p}}{K_{r}}\cdot \frac{\rho_{p}}{\rho_{r}}$$

where only remains to determine what value to assign to the quotient of resistivities *ρ_p_*/*ρ_r_* keeping in mind that they represent equivalent resistivities. For a truly homogeneous soil, *ρ_r_* and *ρ_p_* correspond to the actual resistivity and thus its ratio is the unit. For a two-layer soil of parameters *ρ*_1_, *ρ*_2_ and *h*, we must distinguish between two possibilities. If *ρ*_1_ > *ρ*_2_, then 1 ≤ *ρ_p_*/*ρ_r_* ≤ *ρ*_1_*/ρ*_2_, therefore an upper bound is obtained when the quotient takes the value *ρ*_1_/*ρ*_2_. Nevertheless, if *ρ*_1_ < *ρ*_2_, then *ρ_p_*/*ρ_r_* ≤ 1 and the upper bound is obtained when *ρ_p_*/*ρ_r_* takes the unit value. Therefore, once the type of two-layer soil is established, the procedure for estimating the maximum step potential as a function of the electrode potential is established as follows:

(3) $$U_{p}=U_{r}\cdot \frac{K_{p}}{K_{r}},\;\rho_{1}<\rho_{2}\;U_{p}=U_{r}\cdot \frac{K_{p}}{K_{r}}\cdot \frac{\rho_{1}}{\rho_{2}},\;\rho_{1}>\rho_{2}$$

The determination of the type of two-layer soil is important only in the case that *ρ*_1_ > *ρ*_2_. If as is often the case, the soil has initially been assumed to be homogeneous with resistivity *ρ_s_*, a measurement of the resistance *R* of the electrode by some indirect procedure will provide us not only *U_r_* = *I·R* but also will give us the value of the equivalent resistivity *ρ_r_* = *R*/*K_r_*. Thus, from the expressions (3) results

(4) $$U_{p}=I\cdot R\cdot \frac{K_{p}}{K_{r}},\;\rho_{1}<\rho_{2}\;U_{p}=I\cdot R\cdot \frac{K_{p}}{K_{r}}\cdot \frac{\rho_{s}}{\rho_{r}},\;\rho_{1}>\rho_{2}$$

where the surface resistivity *ρ_p_* in (1) or (2) has been replaced by the equivalent soil resistivity *ρ_s_* estimated in the design and construction stage of the grounding system.

## 3. Preliminary Validation of the Procedure

In this section, the step potentials generated by two types of grounding electrode will be calculated by means of numerical experiments. As input data, the values of the *K_r_* and *K_p_* coefficients will be assumed known, since it will be admitted that they form part of the specifications of the electrodes. The two-layer structure of the soil will also be known, although the expression (4) will also be applied, evaluating *ρ_r_* and *ρ_s_* from the data when necessary. For the validation purposes, the response of the electrode to the injection of a current *I* will be simulated, obtaining the potentials *U_r,sim_* and *U_p,sim_*, according to the specifications of the electrode. By applying the proposed method using the expressions (3) or (4), the calculated step potential *U_p,calc_* will be obtained and is compared with the simulated *U_p,sim_* which would correspond to the really measured potential. For the simulation purposes, a specific software developed by the authors has been used here in which a generic electrode buried in a multilayered soil is excited by a fault current. The grounding resistance and electric potentials are obtained from the currents leaked from the electrode to the ground, after numerically solving the Maxwell equations [11]. The results are summarized in Table 1, while the details of the calculations are shown below.

This section may be divided according to the type of electrode and the type of soil it is buried into. It should provide a concise and precise description of the numerical experiments results, their interpretation as well as the conclusions that can be drawn. 

### 3.1. Vertical Rod

We consider first a vertical rod of longitude *L* = 1 m and radius *r* = 0.005 m, buried at 0.5 m. The maximum step potential is specified as the electrical potential difference between two points that are 1 m apart, the point closest to the electrode being 1 m from the vertical that contains the electrode itself. The coefficients *K_r_* and *K_p_* can be easily calculated resulting in values *K_r_* = 0.828 m^−1^ and *K_p_* = 0.043 m^−1^, where *K_p_*/*K_r_* = 0.052. We will assume for simplicity that the injected current is *I* = 1 A. Four different types of soil are going to be considered.

#### 3.1.1. Type 1

Two-layer soil *ρ*_1_ = 200 Ωm, *ρ*_2_ = 100 Ωm and *h* = 3 m. With this data, *U_r,sim_* = 162.27 V and *U_p,sim_* = 8.37 V are obtained. By taking *ρ_s_* = 200 Ωm and *ρ_r_* = *U_r,sim_*/*K_r_* = 196.03 Ωm, the step potential calculated from (4) results *U_p,calc_* = 8.61 V.

#### 3.1.2. Type 2

Two-layer soil *ρ*_1_ = 100 Ωm, *ρ*_2_ = 200 Ωm and *h* = 3 m. With this data, *U_r,sim_* = 85.06 V and *U_p,sim_* = 4.35 V are obtained. By applying (3), it results *U_p,calc_* = 4.42 V.

#### 3.1.3. Type 3

Two-layer soil *ρ*_1_ = 2000 Ωm, *ρ*_2_ = 100 Ωm and *h* = 3 m. With this data, *U_r,sim_* = 1581.10 V and *U_p,sim_* = 81.34 V are obtained. By taking *ρ_s_* = 2000 Ωm and *ρ_r_* = *U_r,sim_*/*K_r_* = 1910.01 Ωm, the step potential calculated from (4) results *U_p,calc_* = 86.09 V.

#### 3.1.4. Type 4

Two-layer soil *ρ*_1_ = 100 Ωm, *ρ*_2_ =2000 Ωm and *h* = 3 m. With this data, *U_r,sim_* = 95.65V and *U_p,sim_* = 4.51 V are obtained. By applying (3), it results *U_p,calc_* = 4.97 V.

### 3.2. Complex Electrode

**Figure 1:**
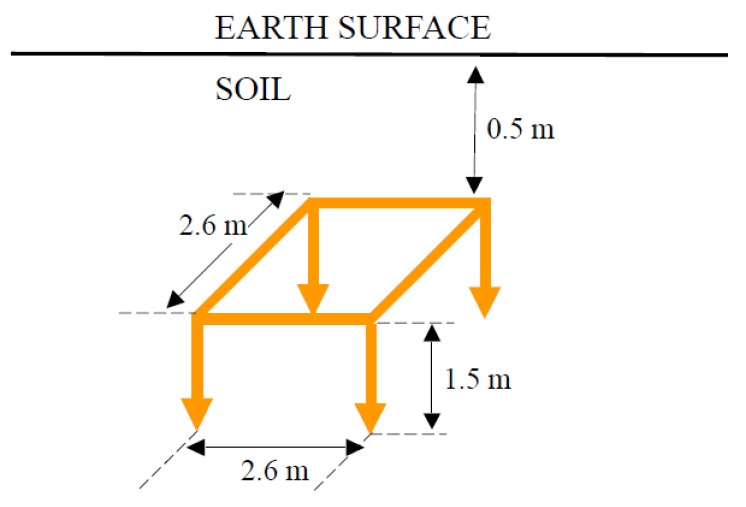
The complex electrode considered as a second example for validation.

**Figure 2:**
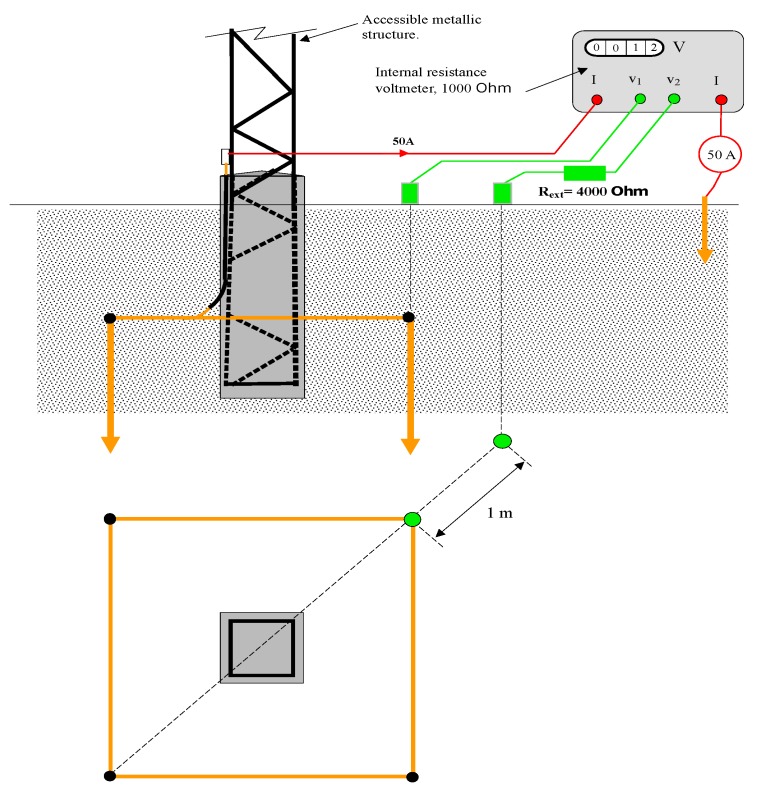
Step potential measurement for the grounding electrode of a power transmission line tower.

Horizontal square frame type electrode of 2.6 m side with vertical rods of 1.5 m length in the vertices and conductors of radius *r* = 0.005 m form the whole set buried 0.5 m from the surface, as shown in Figure 1. Such an electrode is part of the grounding system of a transmission tower, as shown in Figure 2. This figure is a schematic representation of a transmission tower with the grounding electrode of Figure 1. The step potential measurement system requires a device that injects current into the grounding electrode, which returns to through another remote electrode. The potential difference is measured with a high impedance voltmeter. The maximum step potential is specified as the existing electric potential difference between two points of the horizontal frame diagonal separated by a distance of 1 m, the point closest to the electrode being located on the vertical of any of its vertices as is also shown in Figure 2.

The coefficients *K_r_* and *K_p_* are calculated, resulting in the values, *K_r_* = 0.126 m^−1^ and *K_p_* = 0.028 m^−1^ from which *K_p_*/*K_r_* = 0.226 is obtained.

#### 3.2.1. Type 1

Two-layer soil *ρ*_1_ = 200 Ωm, *ρ*_2_ = 100 Ωm and *h* = 3 m. With this data, *U_r,sim_* = 22.14 V and *U_p,sim_* = 5.50 V are obtained. By taking *ρ_s_* = 200 Ωm and *ρ_r_* = *U_r,sim_/K_r_* = 176.31 Ωm, the step potential calculated from (4) results *U_p,calc_* = 5.68 V.

#### 3.2.2. Type 2

Two-layer soil *ρ*_1_ = 100 Ωm, *ρ*_2_ = 200 Ωm and *h* = 3 m. With this data, *U_r,sim_* = 14.67 V and *U_p,sim_* = 2.94 V are obtained. By applying (3), it results *U_p,calc_* = 3.32 V.

#### 3.2.3. Type 3

Two-layer soil *ρ*_1_ = 2000 Ωm, *ρ*_2_ = 100 Ωm and *h* = 3 m. With this data, *U_r,sim_* = 184.87 V and *U_p,sim_* = 52.30 V are obtained. By taking *ρ_s_* = 2000 Ωm and *ρ_r_* = *U_r,sim_/K_r_* = 1471.90 Ωm, the step potential calculated from (4) results *U_p,calc_* = 56.80 V.

#### 3.2.4. Type 4

Two-layer soil *ρ*_1_ = 100 Ωm, *ρ*_2_ = 2000 Ωm and *h* = 3 m. With this data, *U_r,sim_* = 24.90 V and *U_p,sim_* = 3.15 V are obtained. By applying (3), it results *U_p,calc_* = 5.63 V.

### 3.3. The Grounding Electrode of the Balaidos High-Voltage Substation

**Figure 3:**
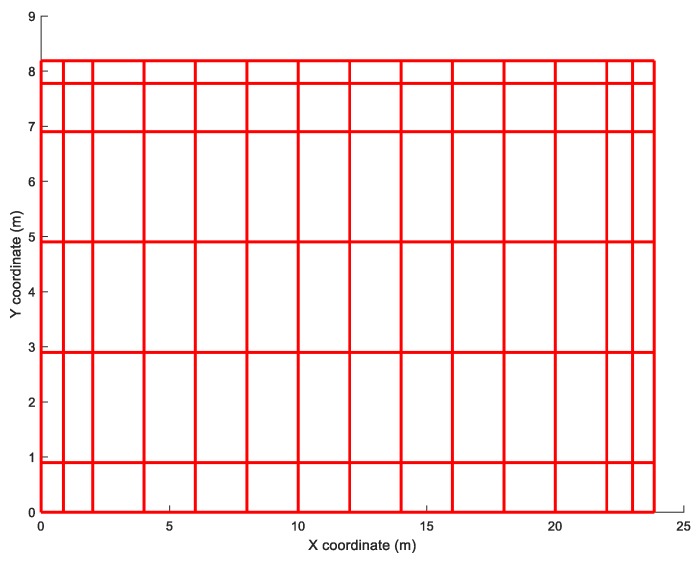
Representation in the XY plane of the grounding electrode of the Balaidos high-voltage substation (Spain).

As the last example of application of the proposed method, the grounding electrode of the Balaidos high-voltage substation, belonging to the Spanish Electric Company *Unión Fenosa* near the city of Vigo (northwest of Spain). The grounding electrode is a mesh of 188 cylindrical conductors (diameter: 11.28 mm) buried at a depth of 80 cm, covering an area of about 200 m^2^. Figure 3 shows the electrode profile in the XY plane. Although the resistivities used in the simulation are fictitious, the electrode is completely real and is buried in a soil of resistivity close to 60 Ωm. For this example, only extreme two-layer soil type 3 and type 4 are considered. The maximum step potential is defined in a similar way to the previous example.

The coefficients *K_r_* and *K_p_* are calculated, resulting in the values, *K_r_* = 0.028 m^−1^ and *K_p_* = 0.004 m^−1^ from which it is obtained *K_p_*/*K_r_* = 0.142.

#### 3.3.1. Type 1

Two-layer soil *ρ*_1_ = 200 Ωm, *ρ*_2_ = 100 Ωm and *h* = 3 m. With this data, *U_r,sim_* = 3.84 V and *U_p,sim_* = 0.62 V are obtained. By taking *ρ_s_* = 200 Ωm and *ρ_r_* = *U_r,sim_*/*K_r_* = 137.14 Ωm, the step potential calculated from (4) results *U_p,calc_* = 0.80 V.

#### 3.3.2. Type 4

Two-layer soil *ρ*_1_ = 100 Ωm, *ρ*_2_ =200 Ωm and *h* = 3 m. With this data, *U_r,sim_* = 4.04 V and *U_p,sim_* = 0.45 V are obtained. By applying (3), it results *U_p,calc_* = 0.57 V.

#### 3.3.3. Type 3

Two-layer soil *ρ*_1_ = 2000 Ωm, *ρ*_2_ = 100 Ωm and *h* = 3 m. With this data, *U_r,sim_* = 20.31 V and *U_p,sim_* = 4.79 V are obtained. By taking *ρ_s_* = 2000 Ωm and *ρ_r_* = *U_r,sim_*/*K_r_* = 739.45 Ωm, the step potential calculated from (4) results *U_p,calc_* = 7.81 V.

#### 3.3.4. Type 4

Two-layer soil *ρ*_1_ = 100 Ωm, *ρ*_2_ = 2000 Ωm and *h* = 3 m. With this data, *U_r,sim_* = 12.14 V and *U_p,sim_* = 0.70 V are obtained. By applying (3), it results *U_p,calc_* = 1.73 V.

Table 1 shows the potential value of the, *Ur,sim*, obtained by simulation, which in this calculation coincides with its resistance since the injected current is 1A. The table also shows the simulated step potentials *Up,sim*, which can be taken as those actually existing in the ground, and their upper levels calculated by (3) and (4) *Up,calc*. The table shows that the best bounds to step potential are calculated for soils where *ρ*_1_ > *ρ*_2_. Otherwise, the bounds are not so good, being able to reach a large difference rate between the true value and the bound, although it can be affirmed that, far from the real value, these bounds represent an absolute limit to the value of the step potential.

**Figure 4:**
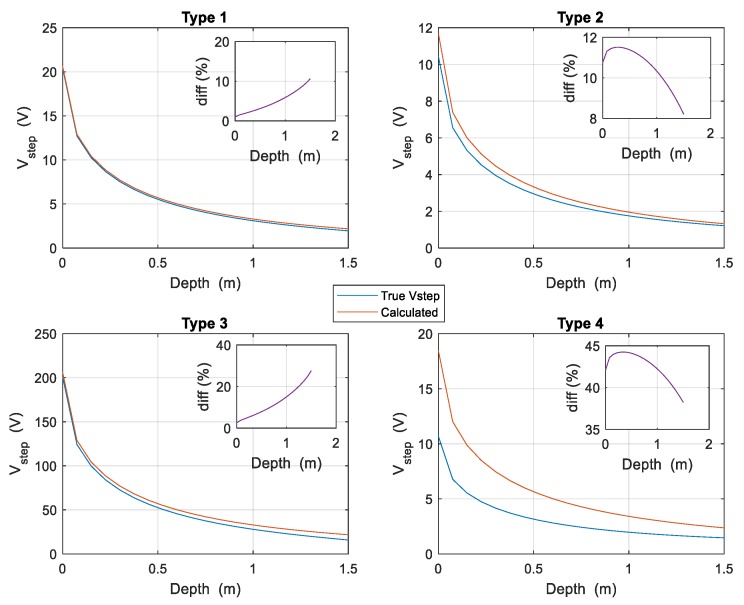
Step potentials created by the complex electrode of Figure 1 for all types of two-layered soils considered, as a function of the burial depth. The upper bound is shown in continuous red line while the real value is in continuous blue line.

For other burial depths of the electrodes, it is necessary to recalculate the values of *K_r_* and *K_p_* and determine the rest of the parameters in order to apply the expressions (4). Figure 4 shows all cases studied for the so-called complex electrode when its burial depth varies from *d* = 0 to 1.5 m, before the electrode crosses the interface between the two layers of the soil. The subfigures show the difference rate between the true value of the step potential and the bound as the electrode varies its burial depth. In Figure 4 it is also observed that the upper bound is very close to the real value of the step potential in when $\rho_{1}>\rho_{2},$ both values being not very far apart. On the other hand, the worst result is obtained when the resistivities involved are very different, and also $\rho_{1}<\rho_{2}$. Nonetheless, it can be verified that the value of the bound for the step potential is always above the true value of this magnitude.

**Figure 5:**
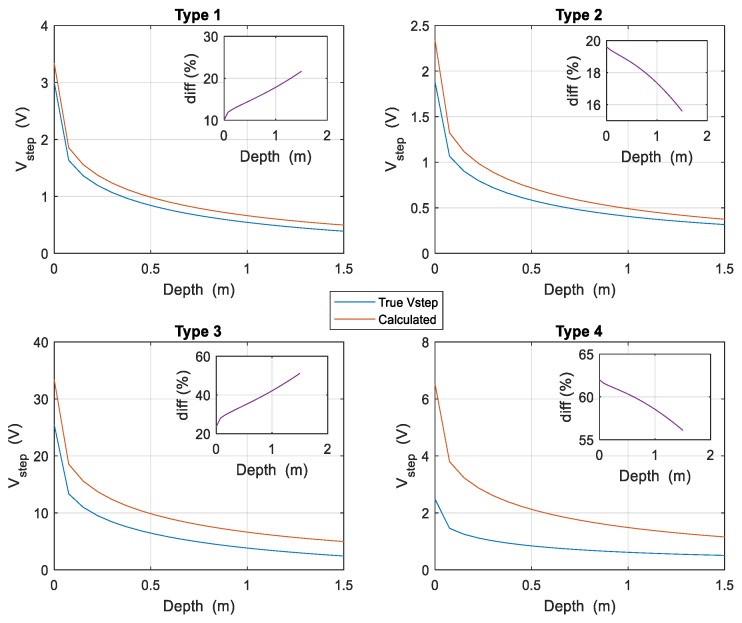
Step potentials generated by the Balaidos grounding electrode for all types of the two-layered soils considered, as a function of the burial depth. The upper bound is shown in continuous red line while the real value is in continuous blue line.

Figure 5 shows all cases studied for the Balaidos grounding grid, where the burial depth of the electrode varies also from *d* = 0 to 1.5 m, before the electrode crosses the interface between the two layers of the soil. The subfigures also show the difference rate between the true value of the step potential and the upper bound as a function of the burial depth. In the same way as described in Figure 4, the upper bound is close to the actual value of the step potential when *ρ*_1_ > *ρ*_2_ while the opposite occurs when *ρ*_1_ < *ρ*_2_ although it is always above the real value of this magnitude.

**Figure 6:**
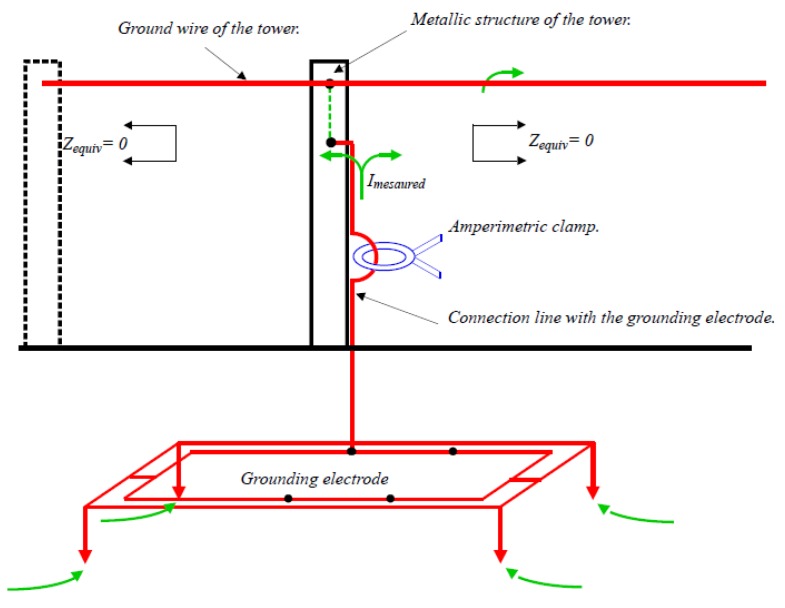
Measurement of the grounding resistance by means of the clamp-on method.

Finally, it is only necessary to comment on the methods to measure the resistance of the electrode, in order to apply the expressions (4). Under general conditions, the measurement is made with a tellurometer using the fall-off-potential method, bearing in mind that the soil is modeled as multi-layered. This method requires that the grounding electrode to be disconnected from the installation. For grounding electrodes that are part of an interconnected system, the clamp-on or stakeless method [12] can be used. It is a fast and reliable non-invasive method. Figure 6 graphically shows a scheme of the clamp-on method. By means of the clamp, an electric current is induced in the grounding system at the same time that the clamp itself measures the resistance of the grounding electrode. 

As a final comment, the more information about the electrode and the electrical structure of the ground, the better the estimate of the upper bound. In cases where the information is incomplete, the bound will be overestimated although it will still be valid for comparison purposes. In these situations, it is possible that this overestimated value of the upper bound exceeds the limits established by the regulatory frameworks, and it is necessary to make direct measurements of the step potentials.

## 4. A Case of Study

As an example of application, a transmission tower, which can be accessed by qualified staff, equipped with a lightning conductor and a non-interconnected grounding system is considered. The tower belongs to the company Union Fenosa Distribucion S.A. and is located in the Spanish region of Consuegra (Toledo). In the construction stage, a homogeneous ground resistivity of 94.95 Ωm was estimated. There is no data available on the multilayer structure of the soil nor on the exact type of electrode used in the installation, but a measurement of the grounding resistance by injecting a current of 5 A with an amperimetric clamp gives a value of *R* = 5.01 Ω and a step potential value of 0.62 V. To compensate for the lack of information on the type of electrode used, the value of the quotient *K_p_*/*K_r_* will be taken as the largest of the values of the electrode database used by the company in similar facilities, which is estimated at *K_p_*/*K_r_* = 0.3. Assuming, for simplicity that *ρ*_1_ = *ρ*_2_, the application of (4) gives a value for the upper bound of the step potential of *U_p,calc_* = 373 V. In order to compare this calculated potential, considered as an upper bound, with what would be obtained in a real situation, it is necessary to know that the installation could generate a fault current of 247.90 A, which taking into account the step potential generated by the current injected could produce a real step potential of *U_p,meas_* = 30.90 V, far below *U_p,calc_*, the upper limit estimated by (4).

## 5. Conclusions

Throughout this paper a procedure to obtain an upper bound to the maximum step potential generated in the ground surface by a grounding electrode excited by a fault current has been presented. The procedure does not replace in any case an accurate assessment of the step potentials but serves to discard the direct measures of these potentials when regulatory standards related to safety are met. The procedure requires knowledge of some characteristic parameters of the electrode *K_r_* and *K_p_*, generally supplied by the manufacturer, although they can be theoretically calculated from the shape and size of the conductors, and some data on the resistivity of the ground, being the most convenient knowledge of the resistivities associated with the two-layer model. Besides this, a direct measurement of the grounding resistance of the electrode is necessary, which can be done by some non-invasive technique such as the clamp-on method. The procedure has been tested on three electrodes in four types of two-layer soils with different resistivity ratios but always assuming that the electrode is located in the upper layer. 

The highest percentage differences between the upper bound and the true value of the maximum step potential are found for high values of the resistivity ratios, especially when *ρ*_1_ < *ρ*_2_. In these situations, the procedure may not serve to discard direct measures of the step potentials. In other situations studied, these differences are relatively small, which allows a greater capacity to comply with regulatory standards, being able to avoid direct measurements. 

In order to study the variation with the burial depth of the electrode of the difference between the simulated step potentials and those calculated with the proposed method, a calculation is made by varying the burial depth up to 1.5 m, and the results are presented in Figure 4 and Figure 5. The Figures also show the variation of the percentage difference between both step potentials.

Finally, a case study has been presented in which, due to the lack of information on the electrode, the upper bound obtained has been greatly overestimated with respect to the true value of the step potential. Although it is unfortunately a common situation, the method always gives a value of the step potential that is always above its real value and thus valid for comparison with regulatory standards.

## Figures and Tables

**Table 1:** Results of the numerical tests for validating the proposed procedure.

Electrode	Two-Layer Soil	*U_r,sim_* (V)	*U_p,sim_* (V)	*U_p,calc_* (V)	Diff (%)
Vertical rod	Type 1	162.27	8.37	8.61	2.8
	Type 2	85.06	4.35	4.42	1.8
	Type 3	1581.10	81.34	86.09	5.8
	Type 4	95.65	4.51	4.97	10.2
Complex	Type 1	22.15	5.50	5.68	3.2
	Type 2	14.67	2.94	3.32	12.8
	Type 3	184.87	52.30	56.80	8.6
	Type 4	24.90	3.15	5.63	78.9
Balaidos	Type 1	3.84	0.62	0.80	22.5
	Type 2	4.04	0.45	0.57	21.1
	Type 3	20.31	4.79	7.81	38.6
	Type 4	12.14	0.70	1.73	59.6

## References

[1] Energy Networks Association (2017). A Guide for Assessing the Rise of Earth Potential at Electrical Installations.

[2] NEN EN 50522:2010. https://infostore.saiglobal.com/en-us/Standards/NEN-EN-50522-2010-799499_SAIG_NEN_NEN_1915943/.

[3] UNE EN 50522:2012. https://infostore.saiglobal.com/en-us/standards/une-en-50522-2012-26163_SAIG_AENOR_AENOR_57403/.

[4] Chen C.X., Xie G.R. (1988). A new formula for calculation of the maximum step voltage in a substation grounding grid. Electr. Power Syst. Res..

[5] Chen L.H., Chen J.F., Liang T.J., Wang W.I. (2008). Calculations of ground resistance and step voltage for buried ground rod with insulation lead. Electr. Power Syst. Res..

[6] Ayodele T.R., Ogunjuyigbe A.S.O., Oyewole O.E. (2018). Comparative assessment of the effect of earthing grid configurations on the earthing system using IEEE and Finite Element Methods. Eng. Sci. Technol. Int. J..

[7] Meliopoulos A.P.S., Patel S., Cokkinides G.J. (1994). A new method and instrument for touch and step voltage measurements. IEEE Trans. Power Deliv..

[8] Nikolovski S., Knezevic G., Baus Z. (2016). Assessment of step and touch voltages for different multilayer soil models of complex grounding grid. Int. J. Electr. Comput. Eng..

[9] Kostic V.I., Raicevic N.B. (2016). An alternative approach for touch and step voltages measurement in high-voltage substations. Electr. Power Syst. Res..

[10] He J., Zhang B., Zeng R. (2015). Maximum Limit of Allowable Ground Potential Rise of Substation Grounding System. IEEE Trans. Ind. Appl..

[11] Faleiro E., Asensio G., Moreno J., Simón P., Denche G., García D. (2016). Modelling and simulation of the grounding system of a class of power transmission line towers involving inhomogeneous conductive media. Electr. Power Syst. Res..

[12] IEEE Guide for Measuring Earth Resistivity (2012). Ground Impedance and Earth Surface Potentials of a Grounding System.

